# Address of the President of the Texas State Medical Association to the Medical Profession

**Published:** 1887-02

**Authors:** T. H. Nott

**Affiliations:** President Texas State Medical Association; Goliad, Texas


### SOCIETY NOTES.

### ADDRESS OF THE PRESIDENT OF THE TEXAS STATE MEDICAL ASSOCIATION TO THE MEDICAL PROFESSION OF TEXAS.

*To the Members of Texas State Medical Association:*

And especially, to those silent members of our profession who, by their silence and inaction, refuse us their countenance, aid and influence, in our undertaking to perfect our organization, in order to purify our profession, elevate its standard, and thereby get possession of the profession in our State, of Medical Legislation—of the Health Department, and all else which of right belongs to us.

By an oversight of mine, this circular is issued one month later than it should be, viz: two months, instead of three months, prior to our annual meeting, as required by Article XII of our By-Laws.

I trust it may prove a virtue in lieu of a fault, as the later the notice the fresher will be the imprint on your minds, and the less the burden upon your memories.

The Texas State Medical Association will meet in Austin on the fourth Tuesday in April, 1887. It is my prescribed duty to urge you to attend. It is not like urging you to say your prayers, for every man has a right to worship at whatever religious shrine he may prefer or select, but it is to urge you to be magnanimous and lend a helping hand to those who are striving for a higher medical standing, which can only be attained through more perfect medical organization. Of course this cannot apply to those who attend each or almost every meeting despite sacrifice and inconvenience, but to those who never attend, or attend only when time, money and opportunity are all propitious. If you cannot come except when it is entirely convenient, come then, and we will give you a hearty welcome, and hope to arouse more interest on your part and make a better Doctor of you. Come one, come all, and let us have such a meeting at our Capital that when we again ask legislation in behalf of the people, and our Legislature attempts to sit down on the good of the people over our shoulders, that the commonest denizens of Austin may realize that that honorable body is attempting to sit higher than its middle will reach. We cannot perfect our organization without labor, expense and personal sacrifice; and without a more perfect organization we cannot occupy that high position of honor, dignity, influence and usefulness to which we are so justly entitled before the law of the State and the bar of public opinion. Then come and help us, one and all.

Especially would I urge upon Chairmen of Sections to come early and bring full reports on their respective sections. This is your opportunity to place yourself on record, not only with the profession of this State, but of the medical world, as a diligent and conscientious worker in your chosen profession; but if you fail to do this, be not chagrined nor dismayed if the profession locate you among the drones—'tis the most charitable nook in which to hide you, while we silently drop over you the veil of brotherly love and charity and forbearance, and appoint some one in your place to do better next year. I trust each member will come with something of interest to report, and prepared to give an intelligent and intelligible experience on what others report; but see to it that your papers are all on the side of science and harmony for the education and elevation of the profession and the promotion of brotherly love within our ranks. If you have hatchets, bury them before you leave home (and don't unearth them when you return). If you *must* write concerning bickerings, dissensions and discord—then don't come at all, but send in your paper that it may be read by caption and referred to our Publishing Committee, whose good judgment and love of the profession we trust would prompt them to decapitate it, and consign it to the waste basket. No member has the right to mar the harmony of our meeting with personal pique or grievance. If your cause of complaint is sufficient, take it before the Judicial Council and have it justly adjudicated as quietly as possible. But let us leave all this off, and come together with loving hearts, and open hands, and show to the world that Doctors can dwell together in peace and unison (for a few days at least).

The character of our Committee of Arrangements is a sufficient guarantee that the programme of our meeting will please all who can be pleased, and strike dumb those critics who make it a point never to be pleased. I have not seen the programme, but take much pleasure in guaranteeing to you a rare treat, and a pleasant and profitable meeting.

Yours truly,

T. H. NOTT, M. D.,

President Texas State Medical Association.

GOLIAD, TEXAS, February 15, 1887.

N. B. It is the duty of all graduates in the State, of creditable medical colleges, to come and join with us. None others, of course, are admitted. T. H. N., M. D., Pres't.

